# Supplementary material for: LMO2 regulates epithelial-mesenchymal plasticity of mammary epithelial cells
Source: Res Sq. 2025 Jul 15:rs.3.rs-7034669. Preprint. [Version 1] doi: 10.21203/rs.3.rs-7034669/v1 (PMC12288518; doi:10.21203/rs.3.rs-7034669/v1)
Supplement: Supplement 1 [file NIHPPrs7034669v1-supplement-1.pdf]

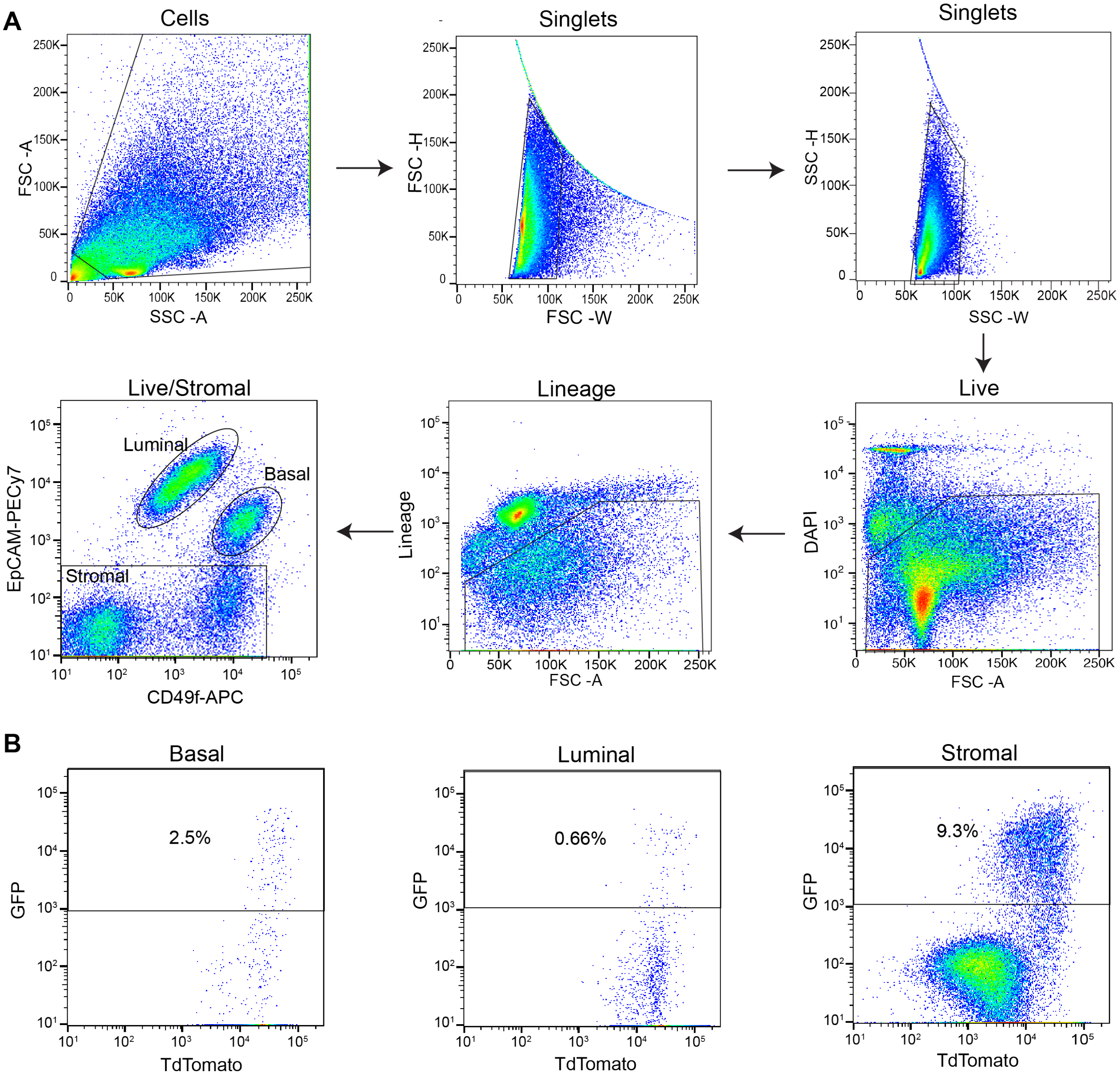

Supplementary Figure 1: Gating strategy for lineage tracing mice *Lmo2^{creERT}/Rosa26^{mTmG}*
Mammary gland from transgenic mice *Lmo2^{creERT2}/Rosa26^{mTmG}* were digested and analyzed with flow cytometry (BD FACSAria). (**A**) Cells were isolated from debris by SSC-A x FSC-A gate and single cells were gated based on the FSC-H x FSC-W and SSC-H x SSC-W. Live cells were gated based on the live/dead cell stain DAPI. Immune cells were gated out with the lineage (lineage -) cocktail (CD45, CD31 and Ter119). Basal epithelial cells were gates based on CD49f^{hi}/EPCAM$^{med/low}$ and luminal cells gated with CD49f^{low}/EPCAM^{hi}. (**B**) Gates for GFP+/TdTomato+ of basal, luminal, and lineage depleted cells.

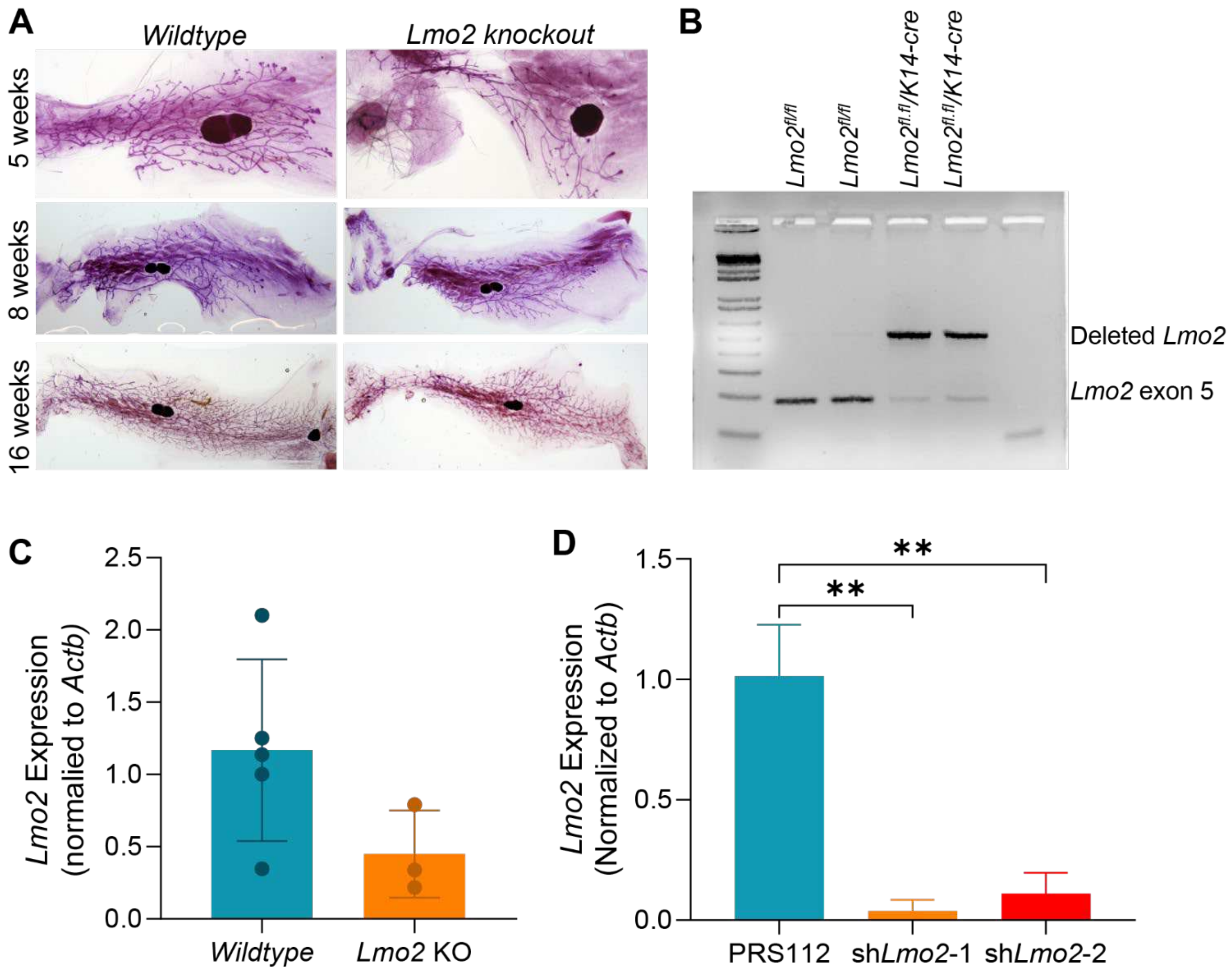

Supplementary Figure 2: *Lmo2* knockout mice genotyping and *Lmo2* knockdown in mouse mammary epithelial cells (**A**) Wholemount images of mammary glands from wildtype *Lmo2*$^{fl/fl}$ and *Lmo2*$^{fl/fl}$*; Krt14-Cre* mice at 5, 8, and 16 weeks of age, stained with Carmine Alum. (**B**) Representative electrophoresis gel showing PCR amplification of *Lmo2* exon 5 in *Lmo2*$^{fl/fl}$ and *Lmo2*$^{fl/lf}$*; Krt14-Cre mice.* (**C**) Quantitative PCR analysis of mammary gland tissue from *Lmo2*$^{fl/fl}$ (n=5) and *Lmo2*$^{fl/fl}$*; Krt14-Cre* (n=3). (**D**) Quantitative PCR analysis of *Lmo2* knockdown in mouse mammary epithelial cells. Statistical significance was calculated using an unpaired t-test. * $p < 0.05$, ** $p < 0.01$, *** $p < 0.001$, **** $p<0.0001$.

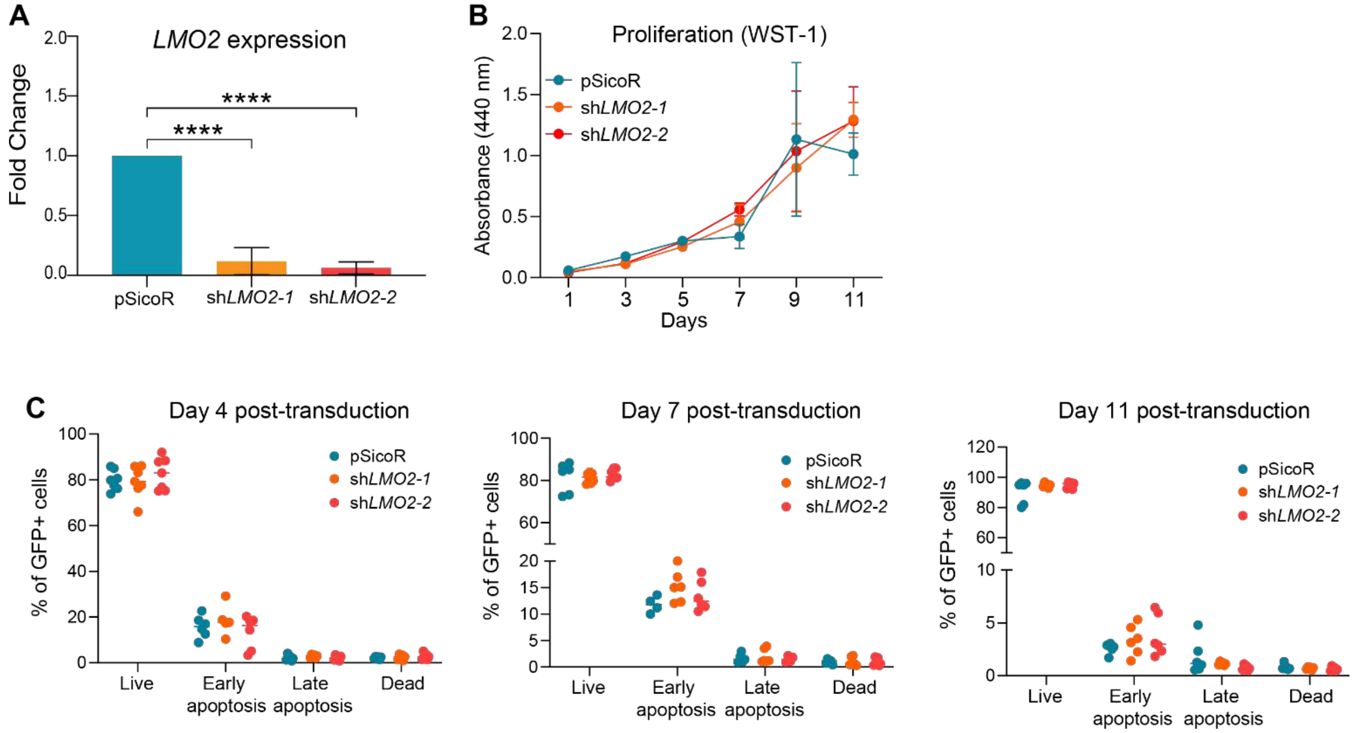

Supplementary Figure 3. Quantification of viability and apoptotic activity in *LMO2* knockdown MCF10A cells (**A**) Quantitative PCR confirming *LMO2* knockdown in MCF10A cells. (**B**) Cell viability assay of control and *LMO2* knockdown MCF10A cells over 11 days (n=3). (**C**) Apoptosis analysis of control and *LMO2* knockdown MCF10A cells at 4, 7, and 11 days post-transduction (n=6), showing proportions of live, early apoptotic, late apoptotic, and dead cells based on GFP+ cells. Data are shown as mean ± SD. Statistical significance was determined by using ordinary one-way ANOVA with multiple comparison test. * $p < 0.05$, ** $p < 0.01$, *** $p < 0.001$, **** $p<0.0001$.

Supplementary Table 2. Antibodies

Antibody	Catalog	Concentration
DAPI	Invitrogen #D1036	1:10,000
PE anti-human CD326	BioLegend #324205	1:100
APC anti-human/mouse CD49f	BioLegend #313616	1:200
PE/Cy7 anti-human CD146	BioLegend #361007	1:200
PE/Cy7 anti-mouse CD326	BioLegend #118216	1:100
PacBlue anti-mouse CD31	BioLegend #102422	1:200
PacBlue anti-mouse CD45	BioLegend #103126	1:200
PacBlue anti-mouse TER-119	BioLegend #116232	1:200
APC Annexin V	BioLegend #640920	1:20
